# Body Mass Index and multidimensional health in Chinese older adults: a moderated mediation analysis of urban–rural residence, physical activity, and depression

**DOI:** 10.3389/fpubh.2025.1650975

**Published:** 2025-09-05

**Authors:** Hao Pan, Ying Hao

**Affiliations:** College of Physical Education, Northwest Normal University, Lanzhou, China

**Keywords:** body mass index, older adults, urban–rural disparities, depression, chronic disease

## Abstract

**Background:**

In the context of China’s accelerated aging, Body Mass Index (BMI) is a critical health metric. However, its health implications are not uniform, being profoundly shaped by structural socioeconomic divides and intricate psychosomatic pathways. This study provides an integrated analysis of BMI’s association with multidimensional health (chronic diseases, functional disability, depression) in Chinese older adults, testing the moderating role of urban–rural residence and the mediating roles of physical activity and depression.

**Methods:**

Data were drawn from the 2018 Chinese Longitudinal Healthy Longevity Survey (CLHLS), including 11,521 older adults. We employed a suite of regression models to assess linear and non-linear associations and a Structural Equation Model (SEM) to delineate mediation pathways.

**Results:**

A higher BMI was associated with a greater chronic disease burden, but not with ADL disability after full adjustment. Crucially, this BMI-disease association was significantly amplified in rural populations, revealing a weaker socio-environmental “buffer” against weight-related health risks. We identified a robust U-shaped relationship between BMI and depression, extending the “obesity paradox” to the psychological domain, with optimal mental well-being observed in the overweight range (BMI ≈ 28.9 kg/m^2^). SEM analysis revealed that depressive symptoms acted as a significant suppressor, partially counteracting the direct adverse association between BMI and chronic diseases.

**Conclusion:**

The health implications of BMI in Chinese older adults are profoundly context-dependent, challenging a one-size-fits-all approach. The findings suggest a dual insight: a psychological “obesity paradox” where moderate overweight status appears protective for mental health, and a socio-environmental moderation where the urban context appears to buffer the physical risks of higher BMI. Mental well-being emerges as a pivotal mediator in the psychosomatic pathway linking body weight to chronic illness. This evidence calls for a paradigm shift toward holistic, differentiated health strategies that integrate mental health support and are tailored to the distinct resource landscapes of urban and rural settings.

## 1 Introduction

China is undergoing a rapid demographic transition toward a deeply aged society. Projections indicate that by 2035, the population aged 60 and over will exceed 400 million, accounting for nearly one-third of the nation’s total population. This profound demographic shift places older adults health at the forefront of the national strategic agenda, as underscored by the Healthy China 2030 initiative and the 2025 National Conference on Aging, which highlighted the urgency of addressing China’s accelerated aging process. Within this context, Body Mass Index (BMI), a core and modifiable metric for assessing weight status, serves as a critical entry point for research. However, its health implications are not uniform, being profoundly shaped by structural socioeconomic divides and intricate psychosomatic pathways. While existing studies have separately examined the roles of BMI, depression, and urban–rural divides in geriatric health, a critical gap remains in understanding how these factors interact within a single, integrated psychosomatic model. Previous work has not simultaneously tested the moderating effect of a socio-environmental context (urban–rural) alongside the mediating psychological and behavioral pathways. This complexity is further amplified by China’s profound urban–rural dichotomy, a structural divide rooted in long-standing socioeconomic policies that systematically shape resource distribution, creating divergent health landscapes: urban older adults often face risks associated with a sedentary lifestyle and high-calorie diets, while their rural counterparts may experience underweight and malnutrition due to physical labor and simpler diets. Crucially, deep-seated disparities in healthcare infrastructure, insurance coverage, and health education exacerbate these differences. Recognizing these intersecting influences, this study utilizes data from the 2018 Chinese Longitudinal Healthy Longevity Survey (CLHLS) to develop an integrated model. Building upon recent work using the CLHLS dataset that has explored BMI trajectories in relation to healthy aging (1) and the mediating role of functional disability in the BMI-depression link (2), aiming to provide a more integrated psychosomatic model. The model is designed to systematically unravel the pathways and boundary conditions through which BMI is associated with multidimensional health outcomes in the older adults—namely, chronic disease burden, Activities of Daily Living (ADL) disability, and depressive symptoms. Specifically, this study examines the mediating roles of physical activity and depression and investigates the moderating effect of the urban–rural divide. The ultimate aim is to provide robust scientific evidence to inform the development of precise, context-aware health interventions for China’s aging population under the “Healthy China 2030” framework.

### 1.1 The complex nexus of BMI and older adults health outcomes

The association between BMI and health in older adults is not linear but is characterized by considerable complexity, most notably the “obesity paradox.” This phenomenon suggests that for older adults, being overweight or even mildly obese may be associated with a lower risk of all-cause mortality. For instance, a meta-analysis of individuals aged 65 and older indicated that the lowest mortality risk occurred at a BMI between 23.0–23.9 kg/m^2^, with risk increasing as BMI fell below 23.0 kg/m^2^ and rising again only when BMI exceeded 33.0 kg/m^2^ (3). Critically, most of these studies were conducted in Western populations, and their findings may not be fully generalizable to the distinct genetic, dietary, and socio-environmental context of China. Further research found this relationship varied by frailty status: among moderately to severely frail older adults, being overweight was protective, whereas high levels of obesity (BMI > 35 kg/m^2^) increased mortality risk in non-frail individuals (4). A systematic review has also supported the existence of the obesity paradox, particularly when older adults present with comorbidities or acute medical conditions (5). Concurrently, obesity is widely recognized as a primary risk factor for chronic diseases. Research shows that compared to their normal-weight peers, obese older adults have significantly higher odds of multimorbidity (≥2 chronic conditions) and a greater prevalence of functional limitations (6). Indeed, obesity is significantly associated with up to 21 distinct cardiometabolic, digestive, respiratory, neurological, musculoskeletal, and infectious diseases, with the risk of complex multimorbidity (≥4 chronic conditions) increasing in a dose-dependent manner (7). Moreover, longitudinal studies suggest that weight fluctuation, in addition to significant gain or loss, can increase all-cause mortality risk compared to maintaining a stable weight, underscoring the importance of weight stability in later life (8).

However, the utility of BMI as a standalone metric in geriatric populations is increasingly being questioned due to its inherent limitations. Crucially, BMI cannot differentiate between body fat and muscle mass. This is particularly relevant in the context of “sarcopenic obesity,” a condition where age-related muscle loss (sarcopenia) coexists with excess body fat, which has complex and often detrimental effects on functional health. Evidence suggests that in older adults, fat mass is positively correlated with adverse health outcomes, while muscle mass is negatively correlated, meaning a single BMI value may not accurately reflect an individual’s true physiological state (9). For example, a study of octogenarians found that a lower body fat percentage and higher appendicular skeletal muscle mass were associated with better functional performance (10). Obesity and aging share numerous pathophysiological mechanisms, including compromised genomic integrity, mitochondrial dysfunction, and chronic inflammation, suggesting that obesity may accelerate the aging process at multiple levels (11). Sarcopenic obesity itself has been robustly linked to accelerated functional decline, increased cardiometabolic risk, and higher mortality (12, 13). This limitation is particularly salient when assessing the link between BMI and functional outcomes like ADL disability and necessitates a more nuanced interpretation that considers body composition.

### 1.2 Moderating and mediating pathways: the role of social context, behavior, and psychology

The health implications of BMI do not occur in a vacuum but are embedded within broader socio-environmental contexts and mediated by individual behaviors and psychological states. In China, the pronounced urban–rural dichotomy serves as a critical moderating variable. Significant disparities in lifestyle, resource availability, and health outcomes exist between urban and rural older adults populations. International studies have also noted the dynamic nature of this health divide; for instance, research in Germany and England-Wales found that while urban areas had higher mortality rates for the 60–79 age group, this trend reversed for those aged 80 and above, where rural areas faced a disadvantage (14). In terms of healthcare, rural older adults in China often face greater accessibility challenges. Compared to urban residents, rural patients with chronic conditions like chronic obstructive pulmonary disease (COPD) report poorer health status and a higher likelihood of forgoing medical care due to financial constraints (15). A broader review of rural health disparities highlights that poorer outcomes in mental, physical, and behavioral health are closely tied to healthcare accessibility (i.e., availability, affordability, and acceptability), which is compounded by socioeconomic factors like poverty, occupation, and education (16). These elements can collectively modulate the impact of BMI on health by shaping lifestyle choices, health literacy, and access to interventions.

Furthermore, the built environment in urban versus rural settings may differentially regulate physical activity levels and, consequently, weight status. Urban environments with high walkability, residential density, and access to public facilities are positively associated with physical activity among older adults, whereas poor infrastructure and safety concerns can be inhibitory (17). Physical activity is a well-established determinant of healthy aging, capable of slowing functional decline (18) and reducing fall risk (19). However, adherence to exercise among the older adults is influenced by a host of factors including program design, social support, and self-efficacy (20), which may vary systematically between urban and rural contexts, thus shaping the trajectory of healthy aging differently (21). Beyond this external moderation, biopsychosocial mechanisms involving physical activity and depressive symptoms may function as critical mediating pathways. Inflammation, for example, has been identified as a partial mediator linking obesity, physical activity, and depression (22). Regular physical activity can lower systemic inflammation and counteract the adverse effects of sedentary behavior through various metabolic and immune pathways, thereby protecting against a range of chronic diseases (23, 24).

Arguably, a pivotal mediating pathway involves psychological health. A complex, bidirectional relationship exists between chronic illness and depression, and research has established that physical symptoms, such as pain, can be a significant mediator in this link (25). Prospective research in Chinese populations has confirmed that functional impairments and chronic pain explain a substantial portion of the association between chronic diseases and depression (26). Moreover, social disconnection and perceived isolation are strong predictors of depression and anxiety in older adults (27). This suggests that an abnormal BMI—whether too high or too low—could exacerbate depressive symptoms by impairing physical function, limiting social participation, and affecting psychological well-being (e.g., self-esteem, body image) (2). In turn, depression can worsen chronic conditions by promoting unhealthy behaviors or through direct neuroendocrine and inflammatory mechanisms (25, 26). This psychological pathway may thus serve as a powerful bridge connecting BMI to chronic disease outcomes.

### 1.3 The present study: research questions and hypotheses

While existing literature has separately examined these factors, there is a scarcity of research that integrates them into a single, comprehensive model to explore their interplay, particularly within the unique Chinese context. To address this gap, this study aims to systematically investigate the relationship between BMI and the health outcomes of chronic disease, ADL disability, and depression, focusing on the moderating role of the urban–rural divide and the mediating effects of physical activity and depressive symptoms. Specifically, this research seeks to answer the following questions: (1) to what extent does BMI demonstrate a non-linear (U-shaped) relationship with depressive symptoms, and at what specific BMI level is psychological well-being optimized? (2) Does the urban–rural context significantly moderate the association between BMI and health outcomes, particularly chronic disease burden, and if so, how does the strength of this association differ between the two groups? (3) Do physical activity and depressive symptoms function as critical mediators in the relationship between BMI and chronic disease burden, and what is the nature of these pathways? Accordingly, the following hypotheses are proposed:

(H1) There is a significant positive association between BMI and the number of chronic diseases in older adults.

(H2) There is a significant positive association between BMI and the risk of ADL disability.

(H3) The relationship between BMI and depressive symptoms is U-shaped, with both low and high BMI levels associated with more severe depressive symptoms.

(H4) Urban–rural status moderates the association between BMI and health outcomes (particularly chronic diseases), with the strength of the association differing between the two groups.

(H5) Physical activity and depressive symptoms mediate the relationship between BMI and the number of chronic diseases, with their direction and strength potentially differing.

## 2 Methods

### 2.1 Data source and sample

This study aims to systematically investigate the association between Body Mass Index (BMI) and multidimensional health outcomes in older adults, with a specific focus on chronic disease burden, Activities of Daily Living (ADL) disability, and depressive symptoms. To this end, the study utilized data from the 2018 wave of the Chinese Longitudinal Healthy Longevity Survey (CLHLS). The survey employs a stratified, multi-stage random sampling methodology covering 22 provinces, autonomous regions, and municipalities across China. This rigorous design, which targets a representative selection of counties and cities within these regions, ensures the sample’s geographical diversity and national representativeness across both urban and rural strata, thereby enhancing the external validity and generalizability of The findings. The timeliness, richness of variables, and extensive coverage of the CLHLS dataset make it an exceptional resource for research on aging and health in China.

A rigorous data screening protocol was implemented to ensure the quality of the analysis. Records with missing values for key variables—including height, weight, ADL assessments, chronic disease diagnoses, gender, age, and depressive symptoms—were excluded. This process yielded a final analytical sample of 11,521 older adults. The sample’s urban–rural composition, with 2,646 urban residents (22.97%) and 8,875rural residents (77.03%), aligns with China’s demographic distribution, underscoring the data’s representativeness. The age of participants in the final analytical sample, reflecting the authentic distribution after data cleaning, ranged from 52 to 117 years (mean = 82.98, SD = 11.06). We made a deliberate methodological and theoretical decision to retain this wide age range. Methodologically, rather than arbitrarily truncating the sample at a specific age (e.g., 65), which can lead to a loss of statistical power and potentially biased estimates, we employed a more robust statistical approach. By including age as a continuous covariate in all our regression models, we precisely control for its linear effects across the full spectrum of later life. Theoretically, this broad range allows us to adopt a life-course perspective, capturing the evolving health dynamics as individuals transition from late-middle age into old age. Finally, this age distribution reflects the authentic demographic profile of the nationally representative CLHLS dataset after rigorous data cleaning. Our approach thus enhances both the statistical rigor and the generalizability of The findings. These sample characteristics provide a robust foundation for the subsequent variable definitions and statistical analyses. The gender distribution was balanced, comprising 6,073 males (52.71%) and 5,448 females (47.29%). The BMI values ranged from 14.35 to 37.56 kg/m^2^, consistent with the weight distribution observed in China’s older adults population. These sample characteristics provide a robust foundation for the subsequent variable definitions and statistical analyses.

### 2.2 Variable measurement

The dependent variables in this study capture three core dimensions of older adults health. First, chronic disease burden (chronic_diseases) was measured as a count of seven prevalent chronic conditions: hypertension, diabetes, heart disease, stroke/cardiovascular disease, dyslipidemia, arthritis, and cholecystitis. These conditions were selected based on their high prevalence in older populations and their established association with weight status. Each condition was coded dichotomously (0 = not diagnosed, 1 = diagnosed), creating a total score ranging from 0 to 7. The sample mean of 1.00 (SD = 1.14) reflects the commonality of multimorbidity in this population. Second, ADL disability (ADL) was assessed using a standard six-item scale consistent with the internationally recognized Katz Index of Independence in ADL, covering bathing, dressing, toileting, indoor transferring, continence, and feeding. Each activity was rated on a three-point scale (1 = completely independent, 2 = some assistance needed, 3 = completely dependent), yielding a total score from 6 to 18. For logistic regression, a binary indicator (ADL_binary) was created where a score of 7 or higher signified disability (coded as 1). This scale has been widely validated for use in Chinese older adults populations and has shown high reliability and validity for assessing functional disability. This scale demonstrated good internal consistency in the sample (Cronbach’s *α* = 0.829). Third, depressive symptoms (depression_score) were measured using a 9-item version of the Center for Epidemiologic Studies Depression (CES-D) Scale, a well-established instrument for assessing depressive symptoms. This condensed version of the original instrument has been extensively validated for screening depressive symptoms among older Chinese adults and has consistently shown good psychometric properties in numerous studies. Items were rated on a 4-point Likert scale and summed to create a total score ranging from 0 to 36, with higher scores indicating greater depressive symptomatology. Positively worded items were reverse-coded to ensure consistent scoring direction. The scale showed acceptable internal consistency in the current study (Cronbach’s *α* = 0.705). This was treated as a continuous variable to preserve nuanced variations in psychological state, with higher scores indicating greater depressive symptomatology.

The primary independent variable was Body Mass Index (BMI), calculated as weight in kilograms divided by height in meters squared (kg/m^2^). To facilitate regression analysis and enhance model comparability, BMI was standardized into a *z*-score (BMI_z) with a mean of 0 and a standard deviation of 1. This transformation helps to mitigate potential issues of multicollinearity, particularly when modeling interaction and quadratic terms, thereby improving parameter interpretability and model stability.

Control variables included age, gender, and urban–rural residence to adjust for potential confounding effects. Age was standardized into a *z*-score (age_z) and included as a continuous variable. Gender (gender) was coded as a binary variable (0 = female, 1 = male). Urban–rural residence (urban) was also coded dichotomously (0 = rural, 1 = urban) and was subsequently employed as a moderator variable to explore its heterogeneous effect on the BMI-health relationship.

Two mediating variables were constructed to elucidate the mechanisms linking BMI to health outcomes. First, exercise consistency (exercise_consistency) was operationalized based on responses to two questions (“Do you exercise regularly now?” and “Did you exercise regularly in the past?”). This variable was coded ordinally: 2 for “yes” to both (consistent exercise), 1 for “yes” to one (partial exercise), and 0 for “yes” to neither (no exercise). This captures not just current behavior but also the stability of physical activity habits over time, which may be a stronger predictor of health outcomes. Second, the previously defined depressive symptoms score was also used as a mediator to investigate the psychological pathway linking BMI to chronic disease burden. To address potential confounding from socioeconomic and family structures, we included three additional sets of control variables. Education level (education) was categorized into four groups: Illiterate/never attended (reference), Primary school, Middle school, and High school or higher. Marital status (marital_status) was coded as: Married (reference), Widowed, and Other (including divorced or never married). Living status (living_status) was categorized as: Living with family (reference), Living alone, and Living in an institution. These variables were included in all subsequent regression and SEM analyses.

### 2.3 Analytical strategy

Prior to model estimation, we conducted diagnostic tests to ensure the validity of our statistical assumptions. We assessed for multicollinearity among the independent variables using the Variance Inflation Factor (VIF). Across all models, the VIF values were well below the conventional threshold of 5, with the highest observed value being 1.32, indicating that multicollinearity was not a significant concern. This ensures the stability and interpretability of the model estimates.

We employed a multi-layered statistical framework aligned with the specific characteristics of the variables and the research objectives. Initially, descriptive statistics were calculated, and differences between urban and rural subsamples were examined using t-tests and chi-square tests. Subsequently, a series of regression models were employed, with the choice of each model carefully justified by the nature of the dependent variable.

To model the number of chronic diseases, a count variable, we first formally tested for overdispersion. The variance of the disease count in the sample (1.30) was found to be substantially greater than its mean (1.00), confirming the presence of overdispersion. Therefore, a standard Poisson model was deemed inappropriate, and a Negative Binomial Regression model was selected to properly account for the data’s distributional characteristics. The model is specified as:

(1) $$\log(\mu )=\beta_{0}+\beta_{1}\cdot {\mathrm{BMI}}_{z}+\beta_{2}\cdot {\mathrm{age}}_{z}+\beta_{3}\cdot \text{gender}+\beta_{4}\cdot \text{urban}$$

To assess the relationship between BMI and the binary ADL disability outcome, a Logistic Regression model was used. The model is specified as:

(2) $$\log(\frac{p_{i}}{1-p_{i}})=\beta_{0}+\beta_{1}\cdot {\mathrm{BMI}}_{z}+\beta_{2}\cdot {\mathrm{age}}_{z}+\beta_{3}\cdot \text{gender}$$

To investigate the potential non-linear relationship between BMI and depressive symptoms, a Linear Regression model incorporating a quadratic term for BMI was employed. A statistically significant quadratic term would suggest a U-shaped or inverted U-shaped relationship, allowing for the identification of an optimal BMI range for mental health. The model is specified as:

(3) $$\text{depressio}\_{\text{score}}_{i}=\beta_{0}+\beta_{1}\cdot {\mathrm{BMI}}_{z}+\beta_{2}\cdot {\mathrm{BMI}}_{z}^{2}+\beta_{3}\cdot {\mathrm{age}}_{z}+\epsilon_{i}$$

To test the moderating effect of urban–rural residence on the BMI-chronic disease association, an interaction term was introduced into the Negative Binomial model. The significance of the interaction term was assessed using a Wald test, and the results were supplemented with scatter plots and fitted curves to visually represent the moderating effect. The expanded model is:

(4) $$\begin{array}{c} \log(\mu_{i})=\beta_{0}+\beta_{1}\cdot {\mathrm{BMI}}_{z}+\beta_{2}\cdot \text{urban}+\beta_{3}\cdot ({\mathrm{BMI}}_{z}\times \text{urban})\\ +\beta_{4}\cdot {\mathrm{age}}_{z}+\beta_{5}\cdot \text{gender} \end{array}$$

These models are formally specified in Equations 1–4. To analyze the mediating effects of exercise consistency and depressive symptoms, a Structural Equation Model (SEM) was utilized. SEM is advantageous as it allows for the simultaneous estimation of multiple pathways and complex causal chains. The primary mediated pathways were designed as: BMI → Exercise Consistency → Chronic Diseases, and BMI → Depression Score → Chronic Diseases. The significance of the indirect effects was determined using bootstrapping with 1,000 resamples to generate 95% confidence intervals; an interval not containing zero indicates a statistically significant mediating effect.

To ensure the reliability of The findings, we conducted a series of robustness and sensitivity analyses. First, to check for the influence of outliers, our primary models were re-run on a trimmed sample that excluded participants with extreme BMI values (BMI < 18.5 kg/m^2^ or ≥30 kg/m^2^). Second, to address potential residual confounding, we conducted sensitivity analyses by re-running our core models with the inclusion of additional, theoretically relevant covariates. Specifically, the moderation model for chronic diseases was re-analyzed controlling for cognitive function, and the U-shaped model for depression was re-analyzed controlling for functional disability status. Consistency in the direction and significance of our key findings across these analyses would strengthen the validity of our conclusions. This integrated analytical framework, combining multiple regression techniques and SEM, was designed to comprehensively capture non-linear associations and test complex moderation and mediation effects, thereby ensuring the depth and reliability of the study’s conclusions.

## 3 Results

### 3.1 Health disparities in urban and rural older adults populations

The analysis began with a descriptive comparison of key characteristics between urban and rural participants. As shown in Table 1, our final sample comprised 11,521 older adults (mean age 82.98 ± 11.06 years; 52.71% male), of whom 2,646 (23.0%) were urban residents and 8,875 (77.0%) were rural residents. Significant urban–rural disparities were observed across nearly all socioeconomic and health indicators. The mean age was statistically comparable between rural (82.97 ± 11.07 years) and urban (83.03 ± 11.00 years) groups, with no statistically significant difference (*p* = 0.814), and the gender distribution was also similar (*p* = 0.165). However, profound differences emerged in socioeconomic status. Rural participants had significantly lower levels of education, with 42.43% being illiterate compared to 19.73% in urban areas (*p* < 0.001). Regarding health indicators, urban older adults exhibited a higher mean BMI (23.43 vs. 22.37 kg/m^2^), a substantially greater chronic disease burden (mean of 1.58 vs. 0.83 conditions), and a higher prevalence of ADL disability (23.20% vs. 14.55%) (all *p* < 0.001). Conversely, rural older adults reported slightly higher average depression scores (10.15 vs. 9.20, *p* < 0.001). Furthermore, urban residents were far more likely to report engaging in consistent exercise (46.86% vs. 21.69%), while the majority of rural participants (65.46%) reported no regular exercise (*p* < 0.001). These marked baseline differences underscore the importance of investigating urban–rural residence as a potential moderator in our subsequent analyses.

**Table 1:** Baseline characteristics of participants by urban–rural residence.

Characteristic	Overall (*N* = 11,521)	Rural (*N* = 8,875)	Urban (*N* = 2,646)	*p*-value
Sociodemographic factors				
Age (years), mean (SD)	82.98 (11.06)	82.97 (11.07)	83.03 (11.00)	0.814
Gender, Male, *n* (%)	6,073 (52.71)	4,710 (53.07)	1,363 (51.51)	0.165
Education level, *n* (%)				<0.001
Illiterate	4,288 (37.22)	3,766 (42.43)	522 (19.73)	
Primary School	3,445 (29.90)	2,702 (30.45)	743 (28.08)	
Middle School	1742 (15.12)	863 (9.72)	879 (33.22)	
High School+	2046 (17.76)	1,544 (17.40)	502 (18.97)	
Marital status, *n* (%)				0.010
Married	5,249 (45.56)	3,984 (44.89)	1,265 (47.81)	
Widowed	5,818 (50.50)	4,535 (51.10)	1,283 (48.49)	
Other	454 (3.94)	356 (4.01)	98 (3.70)	
Living status, *n* (%)				<0.001
With Family	9,133 (79.27)	7,029 (79.20)	2,104 (79.52)	
Alone	1918 (16.65)	1,569 (17.68)	349 (13.19)	
Institution	326 (2.83)	148 (1.67)	178 (6.73)	
Health and lifestyle factors				
BMI (kg/m^2^), mean (SD)	22.62 (4.04)	22.37 (4.03)	23.43 (3.95)	<0.001
Chronic diseases (*n*), mean (SD)	1.00 (1.14)	0.83 (0.98)	1.58 (1.42)	<0.001
ADL Disability, *n* (%)	1905 (16.54)	1,291 (14.55)	614 (23.20)	<0.001
Depression Score, mean (SD)	9.93 (5.30)	10.15 (5.28)	9.20 (5.33)	<0.001
Exercise consistency, *n* (%)				<0.001
No exercise	6,694 (58.10)	5,810 (65.46)	884 (33.41)	
Partial exercise	1,662 (14.43)	1,140 (12.85)	522 (19.73)	
Consistent exercise	3,165 (27.47)	1925 (21.69)	1,240 (46.86)	

**Figure 1:**
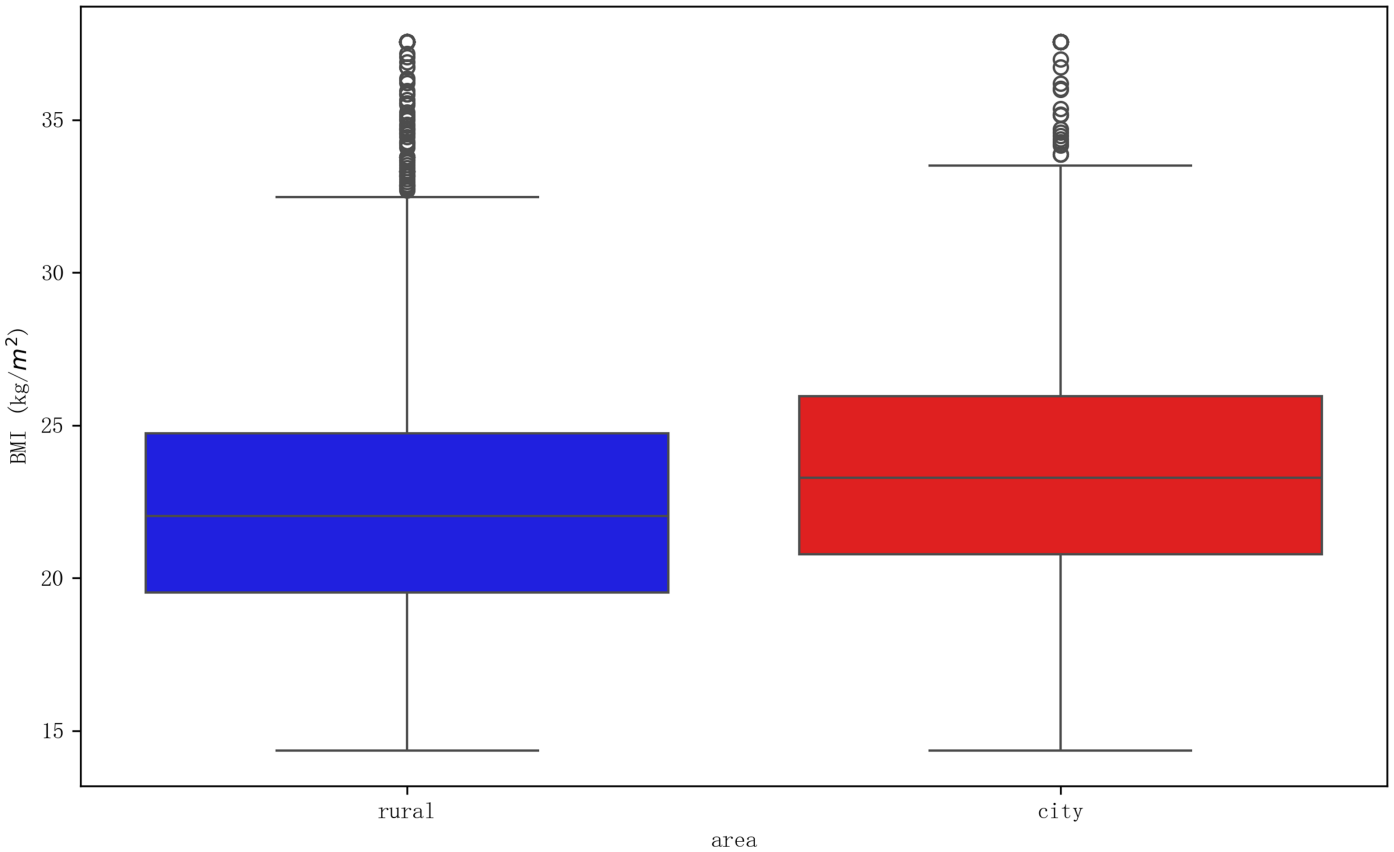
Boxplot of BMI distribution by urban and rural residence. The boxplot visually compares the Body Mass Index (BMI) distributions between rural (left) and urban (right) older adults. The central line in each box represents the median BMI, the box boundaries represent the interquartile range (IQR), and the whiskers extend to 1.5 times the IQR, with individual points indicating outliers. The plot illustrates that urban residents not only have a higher median BMI but also exhibit greater variability in their weight status compared to their rural counterparts.

Figure 1 provides a visual representation of the BMI distributions, illustrating that the median BMI in the urban group was higher than in the rural group. The distribution for urban participants was also more varied, with a notable upward skew, indicating a higher proportion of individuals with high BMI. In contrast, the distribution for rural participants was more concentrated, with a slightly longer lower whisker, suggesting a greater prevalence of individuals with low BMI. These baseline differences highlight the distinct health profiles of urban and rural older adults populations, reflecting how environmental and lifestyle factors may shape health outcomes. The coexistence of higher obesity risk in urban areas and potential undernutrition in rural areas provides a critical context for the subsequent analyses of moderation.

### 3.2 The association between BMI and chronic disease burden: moderation and mediation

**Figure 2:**
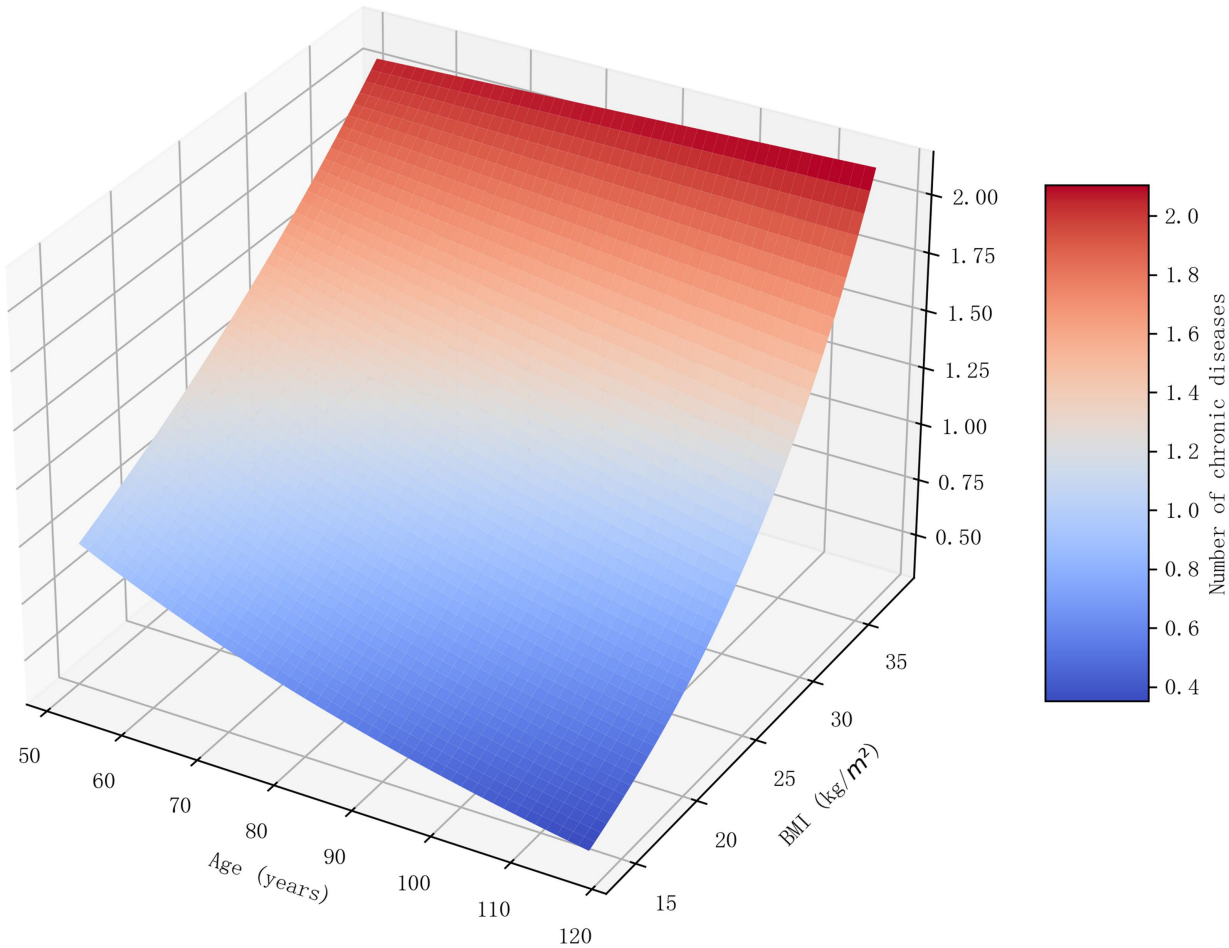
3D surface plot of the combined effect of age and BMI on chronic disease count. This 3D surface plot visualizes the predicted number of chronic diseases based on the interaction between age and BMI, as derived from the negative binomial regression model. The *x*-axis represents age, the y-axis represents BMI, and the *z*-axis (indicated by both height and color intensity) represents the predicted count of chronic diseases. The plot clearly demonstrates that the risk of multimorbidity is highest in the stratum of older individuals with a high BMI, highlighting a compounding risk effect.

To test the association between BMI and chronic disease burden, a negative binomial regression model was employed. As detailed in Table 2, the analysis revealed that a higher BMI was significantly associated with a greater chronic disease burden (RR = 1.244, *p* < 0.001). This finding suggests that for each standard deviation increase in BMI, the expected count of chronic diseases increases by approximately 24.4%. As illustrated in the 3D surface plot in Figure 2, this cumulative risk was most pronounced in the high-age, high-BMI stratum (age > 90, BMI > 30 kg/m^2^), where the predicted number of conditions approached 2.5. The model also revealed independent associations with socioeconomic and living conditions. Notably, living in an institution was associated with a higher number of chronic diseases (RR = 1.230), and having primary or middle school education was linked to a slightly higher disease count compared to being illiterate, possibly reflecting better health awareness and higher diagnosis rates.

**Table 2:** Negative binomial regression of BMI on number of chronic diseases.

Variable	*β* (coef)	Std. err.	*p*-value	RR	95% CI
Main effects					
BMI (*z*-score)	0.218	0.014	<0.001	1.244	[1.210, 1.278]
Age (*z*-score)	−0.085	0.018	<0.001	0.919	[0.888, 0.951]
Gender (Ref: female)					
Male	0.225	0.030	<0.001	1.252	[1.180, 1.330]
Residence (Ref: rural)					
Urban	0.559	0.032	<0.001	1.749	[1.642, 1.861]
Control variables					
Education (Ref: illiterate)					
Primary school	0.076	0.037	0.038	1.079	[1.004, 1.159]
Middle school	0.157	0.046	0.001	1.170	[1.069, 1.279]
High school+	0.062	0.042	0.144	1.063	[0.980, 1.154]
Marital status (Ref: married)					
Widowed	−0.055	0.037	0.137	0.946	[0.879, 1.018]
Other	−0.165	0.085	0.051	0.848	[0.717, 1.001]
Living status (Ref: with family)					
Living alone	0.051	0.040	0.209	1.052	[0.972, 1.139]
In an institution	0.207	0.078	0.008	1.230	[1.055, 1.433]
Intercept	−0.365	0.036	<0.001		

Next, we investigated the moderating role of urban–rural residence. The analysis was extended to investigate the moderating role of urban–rural residence. A significant interaction effect was confirmed (*β* = −0.088, *p* = 0.003; see Table 3), indicating that the relationship between BMI and chronic disease burden is not uniform across these settings. Specifically, the risk increase associated with a higher BMI was more pronounced in the rural population (RR ≈ 1.27) compared to the urban population (RR ≈ 1.17). This suggests that while urban older adults have a higher baseline chronic disease burden, the adverse impact of rising BMI on this burden is more pronounced in rural settings, where healthcare resources may be less equipped to buffer this risk.

**Table 3:** Interaction effect of BMI and urban–rural residence on chronic diseases.

Variable	*β* (coef)	SE	*p*-value
BMI (*z*-score)	0.241	0.016	<0.001
Urban	0.577	0.033	<0.001
BMI_z * Urban	−0.088	0.030	0.003
Age (*z*-score)	−0.085	0.018	<0.001

To delineate the mediating pathways, a Structural Equation Model (SEM) was specified, controlling for age, gender, and key socioeconomic factors. The model demonstrated acceptable goodness-of-fit and revealed a significant positive direct association between standardized BMI and the number of chronic diseases (standardized *β* = 0.193, *p* < 0.001). Alongside this direct effect, two distinct indirect pathways were identified.

First, a modest mediating pathway was observed through exercise consistency, which yielded a small positive indirect effect (*β* = 0.0014). This was characterized by a positive path from consistent exercise to a higher number of chronic diseases (b1 path: *β* = 0.028, *p* < 0.01). In contrast, the second, more substantial pathway involving depressive symptoms proved to be substantially more influential, functioning as a statistical suppression effect (indirect *β* = −0.0095). This complex relationship emerged because a higher BMI was associated with fewer depressive symptoms (a2 path: *β* = −0.074, *p* < 0.001), while greater depressive symptoms were, in turn, strongly linked to a higher chronic disease burden (b2 path: *β* = 0.128, *p* < 0.001). Consequently, this indirect psychological route partially counteracted, or “suppressed,” the direct adverse association between BMI and chronic diseases.

### 3.3 Associations of BMI with ADL disability and depressive symptoms

As presented in Table 4, after controlling for a full set of demographic and socioeconomic variables, BMI_z was no longer a statistically significant predictor of ADL disability (OR = 1.049, *p* = 0.109). Age remained the predominant factor associated with disability (OR = 3.044, *p* < 0.001). Notably, higher education and living alone were associated with lower odds of disability, whereas being widowed and living in an institution were linked to higher odds. Ultimately, this suggests that socioeconomic and living contexts may be stronger correlates of functional disability than weight status itself in this population.

**Table 4:** Logistic regression of BMI on ADL disability.

Variable	*β* (coef)	SE	*p*-value	OR	95% CI
Main effects					
BMI (*z*-score)	0.048	0.030	0.109	1.049	[0.989, 1.112]
Age (*z*-score)	1.113	0.039	<0.001	3.044	[2.822, 3.284]
Gender (Ref: female)					
Male	0.062	0.066	0.342	1.064	[0.934, 1.213]
Residence (Ref: rural)					
Urban	0.687	0.068	<0.001	1.988	[1.737, 2.275]
Control variables					
Education (Ref: illiterate)					
Primary school	−0.275	0.077	<0.001	0.760	[0.654, 0.884]
Middle school	−0.203	0.107	0.059	0.816	[0.662, 1.006]
High school+	−0.467	0.090	<0.001	0.627	[0.526, 0.748]
Marital status (Ref: married)					
Widowed	0.370	0.079	<0.001	1.448	[1.240, 1.690]
Other	−0.077	0.225	0.732	0.926	[0.594, 1.443]
Living status (Ref: with family)					
Living alone	−0.887	0.090	<0.001	0.412	[0.345, 0.491]
In an institution	0.528	0.137	<0.001	1.695	[1.296, 2.217]
Intercept	−2.189	0.079	<0.001		

**Figure 3:**
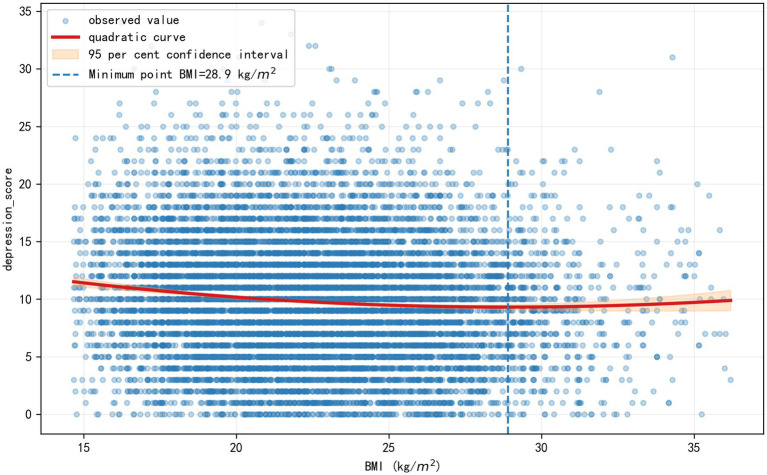
The U-shaped relationship between BMI and depressive symptoms. The curve illustrates the predicted depression score derived from the linear regression model (Table 5), adjusted for all demographic and socioeconomic covariates. The nadir of the curve, representing the point of lowest predicted depressive symptomatology, corresponds to a BMI of approximately 28.9 kg/m^2^, which falls within the overweight range.

To investigate the association between BMI and psychological health, we employed a linear regression model with a quadratic term for BMI_z. The results, detailed in Table 5, confirmed a significant and robust U-shaped relationship. The high statistical significance of both the negative linear term (*β* = −0.498, *p* < 0.001) and the positive quadratic term (*β* = 0.158, *p* < 0.001) provides strong support for this non-linear association. As visualized in Figure 3, this U-shaped curve reaches its nadir, representing the lowest predicted depression score, at a z-score of 1.58, which corresponds to a BMI of approximately 28.9 kg/m^2^. This suggests that while low BMI is associated with elevated depressive symptoms, a moderate level of overweight may be linked to better psychological well-being in this older adults population. The model also powerfully demonstrated the role of social determinants in mental health. Higher education was strongly associated with fewer depressive symptoms, while being widowed, living alone, or living in an institution were all significant predictors of greater depressive symptoms.

**Table 5:** Linear regression on depressive symptoms (CES-D score).

Variable	*β* (coef)	SE	*t*-value	*p*-value
Main effects				
BMI (*z*-score)	−0.498	0.056	−8.829	<0.001
BMI (*z*-score)^2^	0.158	0.031	5.126	<0.001
Age (*z*-score)	0.091	0.063	1.446	0.148
Gender (Ref: female)				
Male	−0.660	0.109	−6.066	<0.001
Residence (Ref: rural)				
Urban	−0.552	0.123	−4.479	<0.001
Control variables				
Education (Ref: illiterate)				
Primary school	−0.814	0.130	−6.256	<0.001
Middle school	−1.333	0.171	−7.778	<0.001
High school plus	−0.346	0.148	−2.333	0.020
Marital status (Ref: married)				
Widowed	0.406	0.132	3.068	0.002
Other	0.885	0.296	2.993	0.003
Living status (Ref: with family)				
Living alone	1.087	0.143	7.599	<0.001
In an institution	1.774	0.301	5.885	<0.001
Intercept	9.582	0.129	74.298	<0.001

### 3.4 Robustness and sensitivity analyses

A series of analyses confirmed the robustness of the study’s main findings. First, the U-shaped relationship between BMI and depressive symptoms remained significant after excluding participants with extreme BMI values (<18.5 or ≥30 kg/m^2^). An analysis using this trimmed sample also revealed a significant interaction between BMI and urban–rural status (*β* for interaction term = −0.121, *p* = 0.009), indicating the adverse psychological association of high BMI was more pronounced in the rural population. Furthermore, sensitivity analyses demonstrated that the core findings were robust to potential residual confounding. The significant moderating effect of urban–rural residence on the BMI-chronic disease link remained after controlling for cognitive function (β for interaction term = −0.090, *p* = 0.002), and the U-shaped BMI-depression relationship remained highly significant after adjusting for functional disability status (β for BMI_z^2^ = 0.171, *p* < 0.001). Collectively, these results indicate that the study’s conclusions are not substantially driven by outliers or by confounding from cognitive status or physical disability.

## 4 Discussion

This study, utilizing data from the 2018 Chinese Longitudinal Healthy Longevity Survey (CLHLS), systematically investigated the complex associations between Body Mass Index (BMI) and multidimensional health outcomes in older adults. By employing an integrated analytical framework that incorporates both moderation and mediation effects, the research not only validated the presupposed theoretical pathways but also provided robust empirical support for advancing both theoretical understanding and practical interventions in geriatric health.

### 4.1 The non-linear relationship between BMI and geriatric depression: extending the obesity paradox

A central finding of this investigation is the significant U-shaped relationship identified between BMI and depressive symptoms, which supports our third hypothesis. This discovery is particularly noteworthy, as it directly engages with the “obesity paradox” phenomenon. The nadir of the depression risk curve was observed in the overweight range, at a BMI of approximately 28.9 kg/m^2^. This finding not only directionally aligns with previous meta-analyses that reported a reduced mortality risk for moderately overweight older adults (3) but also innovatively extends the obesity paradox from a purely physiological dimension to a psychological one. The specific turning point identified in the study is highly consistent with values reported in recent research on Chinese populations (28–30). Such consistency across studies lends considerable weight to the potential protective effect of “moderate overweight” on mental well-being in later life.

While this U-shaped relationship is statistically robust, it is important to contextualize this finding by acknowledging that the magnitude of the “protective” association is modest. However, it can be argued that that the primary contribution of this finding lies not in its clinical effect size, but in its significant theoretical implication. By demonstrating this non-linear pattern, the study challenges the prevailing linear, risk-oriented view of BMI in mental health and suggests that the conversation must shift from a simple “lower is better” paradigm to a more nuanced, optimal-range perspective. The value of this finding is therefore in conceptually extending the “obesity paradox” into the psychological realm and highlighting that the mechanisms linking weight to mental health may be fundamentally different from those linking it to cardiometabolic diseases.

The mechanism underlying this non-linear association may be twofold. On one hand, it could stem from the unique physiological needs of older adults, where modest fat reserves may help buffer against catabolic diseases and frailty (11), consequently alleviating the psychological distress associated with these conditions. On the other hand, it might be linked to sociocultural factors, such as the traditional Chinese perception of plumpness as a sign of fortune and well-being. At the same time, our explanation for the elevated depression risk at low body weights is consistent with multiple studies linking malnutrition to an increased risk of depression in the older adults (31–33), further refining the mechanistic understanding of this U-shaped relationship.

### 4.2 The urban–rural divide: socio-environmental moderation of BMI’s health effects

Confirming Hypothesis 1, the study found that a higher BMI is significantly associated with a greater number of chronic diseases. However, a more complex picture emerged for physical function: while a bivariate correlation supported Hypothesis 2, the association between BMI on ADL disability became non-significant in our fully adjusted model. This crucial finding suggests that while obesity is generally linked to functional limitation (6), its direct predictive power is diminished when socioeconomic context is accounted for. This prompts a deeper reflection on two interconnected factors. First, the inherent limitations of the BMI metric itself, which cannot differentiate between fat and muscle mass, are particularly relevant for older adults, whose functional status is more dependent on body composition (12, 13); indeed, some studies find that the ratio of lean to fat mass is a superior predictor (9). Second, and more importantly, the results suggest that social determinants of health (34)—such as education, marital status, and living arrangements—may be more potent correlates of functional independence than BMI alone.

Beyond these direct associations, the findings validated Hypothesis 4 by revealing that the urban–rural context significantly moderates the BMI-chronic disease relationship, a finding highly consistent with widely reported health differentials (14, 15). Specifically, the adverse association of rising BMI was more pronounced among rural older adults. This disparity may be attributable to what we theorize as a relatively weaker “healthcare buffer “in rural areas, encompassing poorer healthcare infrastructure, lower health literacy, and different social support networks (16). Our finding provides empirical weight to this theoretical assertion by demonstrating a tangible consequence: the same biological risk factor (rising BMI) translates into a greater realized health burden in the rural context. This suggests that urban healthcare systems may act as a crucial “buffer,” effectively mitigating some of the physiological risks associated with higher BMI. This buffer is likely composed of tangible elements such as more comprehensive health insurance schemes, better-funded pension systems that improve affordability, and a higher density of specialized medical facilities and personnel, enabling superior disease management and more accessible health education. In rural settings, where such buffers appear are weaker, the link between BMI and chronic disease remains more direct and potent. Therefore, our moderation finding illuminates the pathways through which structural inequality may materially shape individual health trajectories. In contrast, although urban older adults may have a higher baseline prevalence of chronic diseases due to their lifestyles (7), the association of BMI with health appeared attenuated, likely due to more accessible healthcare and management resources (15). This urban–rural moderation, combined with the finding on ADL disability, powerfully highlights that structural societal factors are pivotal in shaping health outcomes, underscoring the necessity for geographically tailored intervention strategies.

### 4.3 The mediating roles of physical activity and depression: a psychosomatic pathway

**Figure 4:**
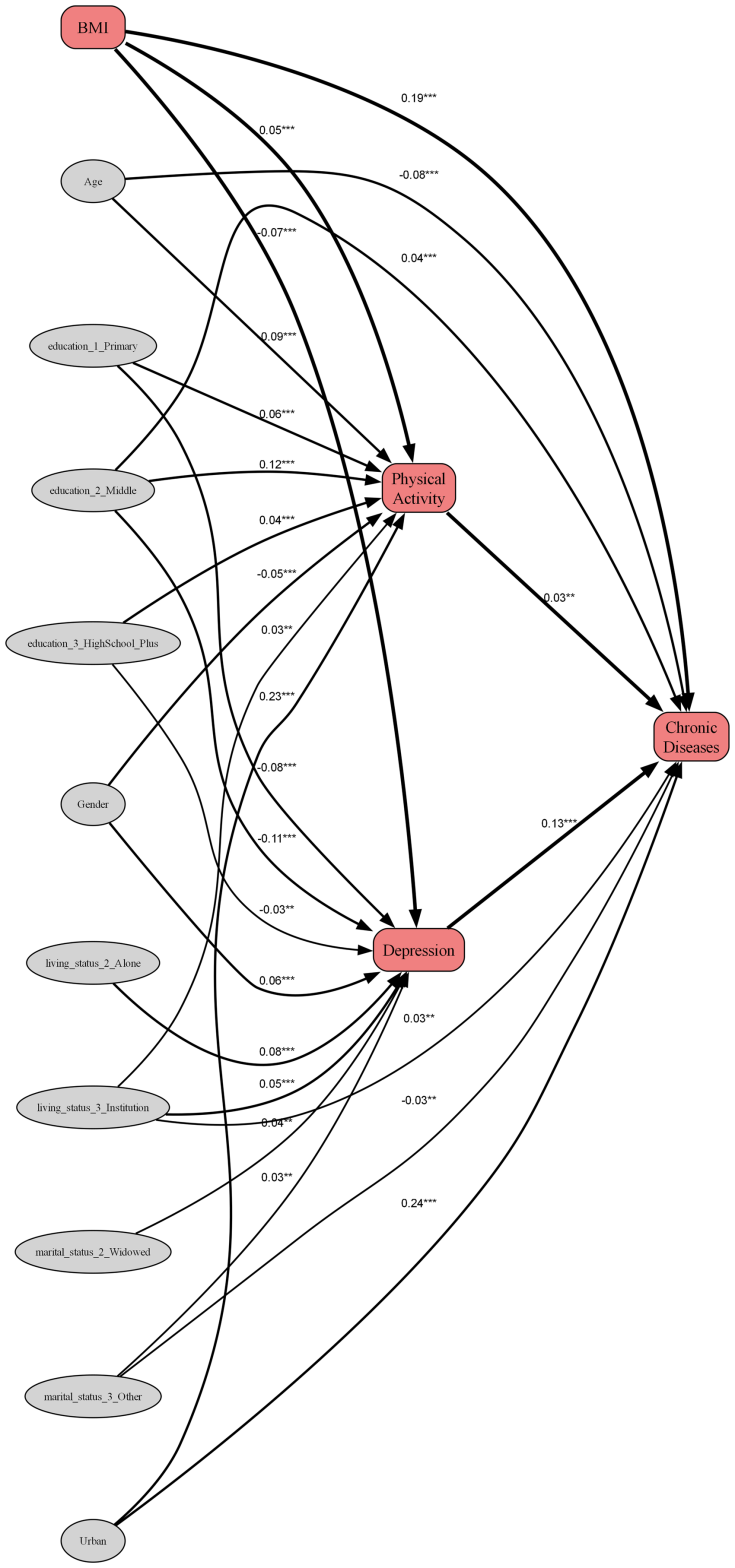
Path diagram of the structural equation model. Standardized path coefficients are shown. The model demonstrates that the direct positive effect of BMI on chronic diseases (*β* = 0.193) is partially counteracted by a significant indirect suppression effect operating through depression (indirect effect *β* = −0.0095). Paths from control variables (age, gender, urban–rural residence, education, marital status, and living status) were included in the model but are omitted from the diagram for visual clarity. ***p < 0.001, **p < 0.01, *p < 0.05.

The SEM analysis confirmed Hypothesis 5 by uncovering the mediating mechanisms of physical activity and depression in the relationship between BMI and chronic conditions. This deepens our understanding of integrated psychosomatic health management in old age. Although the positive mediating effect of physical activity was modest, its statistical significance is in line with findings from other studies suggesting that physical activity promotes health by improving inflammatory status (22, 24)or mitigating the risks of a sedentary lifestyle (21). However, the path from exercise to chronic diseases in our SEM model (Figure 4) was positive, which appears counter-intuitive. This should not be interpreted as exercise being harmful. A more plausible explanation, given our cross-sectional data, is the potential for reverse causality: older adults who have already been diagnosed with chronic conditions are often medically advised to engage in regular physical activity. Therefore, this pathway likely reflects a health-seeking behavioral response to a pre-existing condition, rather than a causal effect of exercise on disease incidence.

Notably, the negative mediating effect of depressive symptoms was substantially more pronounced. This finding corroborates an extensive body of literature that emphasizes the complex, bidirectional interactions between chronic diseases and depression, where factors such as pain, functional impairment, and social isolation play key mediating roles (25–27). The results suggest that mental health is not merely a passive outcome of physical illness; rather, it appears to be an active component in the pathways linking body status to the progression of physiological chronic diseases, potentially through its effects on health behaviors, neuroendocrine balance, and even immune function. This framing carefully avoids causal claims while still highlighting the variable’s importance. This establishes mental health as a pivotal nexus in mind–body interactions, providing a strong scientific rationale for integrating mental health interventions into weight management and chronic disease prevention programs under China’s “Healthy China” initiative.

A key contribution of this study is the simultaneous revelation of a psychological pathway (mediation by depression) and a contextual determinant (moderation by residence). When viewed together, these findings paint a more intricate picture. The moderation analysis showed the BMI-disease link is stronger in rural areas. While the mediation model established depression as a general pathway, it is plausible to speculate that this very psychosomatic pathway (BMI → Depression → Disease) is itself amplified in rural settings. This suggests a profound and complex form of health disparity. Rural elders appear to face a double burden: not only are they more vulnerable to the direct physical consequences of abnormal BMI due to a weaker healthcare buffer, but they may also lack access to the resources needed to buffer the psychological consequences. The scarcity of both specialized chronic disease management and accessible mental health services in rural areas could create a vicious cycle, where the physiological stress of weight-related illness and the psychological distress of depression reinforce one another with fewer opportunities for effective intervention.

### 4.4 Theoretical contributions and practical implications

The theoretical contributions of this research are multidimensional. First, by extending the “obesity paradox” from the domain of physiological mortality (35) to psychological health, this research contributes a novel conceptual element to the theoretical reframing of health indicators for the older adults. Second, by quantifying the moderating effect of the urban–rural divide, it elucidates how the social environment can reshape the health consequences of a biological marker like BMI, opening new avenues for integrating health sociology and epidemiology. Third, the SEM analysis establishes the core mediating role of depression in the psychosomatic pathway, offering a methodological paradigm for interdisciplinary research.

On a practical level, this study provides a scientifically rigorous and actionable blueprint for geriatric health management in China. Furthermore, these findings offer broader insights for global health policy. Many nations are facing similar challenges of rapid aging and urban–rural health disparities. The key takeaways—that psychological well-being is intertwined with physical health metrics like BMI, and that “healthy” weight standards may need to be context- and age-specific—are universally relevant. This calls for a global shift toward integrating mental health services into standard geriatric care and developing health policies that are sensitive to socioeconomic and geographical contexts. The findings suggest three key strategies based on The findings. First, the development of differentiated weight management strategies for older adults, recognizing that the optimal BMI for minimizing depression differs from that for minimizing chronic disease. Second, the integration of mental health interventions into weight management programs, as depression is a key mediator affecting health outcomes. Third, the creation of tangible, visual health education tools. The interaction plots and U-shaped curves from the study are not just academic figures; they can be transformed into intuitive public health materials. For instance, primary care providers could use a simple, color-coded “Health Triangle” chart that visually connects BMI (body), depression score (mind), and chronic disease count (illness). For rural populations, this could be adapted into a laminated flip chart for use by community health workers during home visits. Such tools would translate complex statistical findings into actionable, personalized advice, empowering older adults to see weight management not just as a physical task, but as a proactive strategy for preserving both mental and physical well-being.

### 4.5 Limitation

Despite the contributions of this study, several limitations should be acknowledged, which in turn suggest avenues for future research. First, the cross-sectional design of the study inherently restricts our ability to make definitive causal inferences. For instance, while we modeled depression as a mediator between BMI and chronic disease, it is equally plausible that the burden of chronic illness could influence both mental health and weight status over time. The relationships we modeled, particularly the mediational pathways, are based on a well-established theoretical framework, but the associations could be bidirectional. Longitudinal studies are essential to establish the temporal sequence and causality of these complex interactions.

Second, the study is subject to limitations related to variable measurement. The use of BMI as the primary metric for weight status, while standard, does not differentiate between fat mass and muscle mass. This is particularly relevant in older adults, where “sarcopenic obesity”—a condition of low muscle mass coexisting with high fat mass—is a significant health concern that our BMI measurement cannot capture. This limitation may have contributed to the non-significant association observed between BMI and ADL disability after full adjustment, as functional capacity is often more closely tied to body composition than to BMI alone. Furthermore, the self-reported nature of chronic disease diagnoses could be subject to recall bias, wherein individuals with higher health literacy or more frequent contact with the healthcare system may report their conditions more accurately than their counterparts.

Third, the potential for selection bias warrants consideration. Although the CLHLS is designed to be a nationally representative survey, our analytical sample was derived after excluding participants with missing data on key variables. This process could introduce a selection bias if the pattern of missingness was not completely at random. For example, if older adults who were sicker, more socially isolated, or more cognitively impaired were disproportionately likely to have incomplete records, our findings might represent a slightly healthier or more robust subset of the older population, potentially underestimating the strength of some associations.

Finally, while our sensitivity analyses confirmed the robustness of key findings, the models did not include all potential confounders. For instance, the CLHLS dataset lacked detailed data on chronic pain or specific dietary patterns (36), which are known to mediate or confound the relationships between weight, chronic illness, and depression. Similarly, the *R*^2^ value for our depression model indicates that other unmeasured variables, such as social support networks or specific life events, are important predictors of geriatric depression. Building on our findings, a crucial next step would be to employ multi-group structural equation modeling. This advanced technique would allow for an explicit test of moderated mediation—that is, whether the psychosomatic pathway linking BMI, depression, and chronic disease operates differently in urban versus rural contexts, offering a more granular and actionable understanding.

## 5 Conclusion

This study, drawing upon a large, nationally representative sample of Chinese older adults, offers a nuanced perspective on the multifaceted relationship between body weight and health, suggesting the implications of BMI in later life are neither linear nor uniform. A key finding suggests a U-shaped association between BMI and depressive symptoms, with the lowest psychological distress observed in the overweight range. This potentially extends the conventional “obesity paradox” from a physiological concept to the domain of mental well-being, which may challenge a universal, one-size-fits-all standard for healthy weight in geriatric populations.

Furthermore, the research suggests that the link between BMI and chronic disease appears to be significantly moderated by the socio-environmental context. The stronger association found in rural populations suggests that health outcomes are shaped by a “double burden”: a weaker healthcare buffer against the physical risks of high BMI and potentially fewer resources to mitigate the associated psychological distress. This is further supported by our finding that functional ability was more strongly linked to socioeconomic context than to BMI itself. The analysis also points toward a potentially critical psychosomatic pathway, revealing that depressive symptoms may act as a significant mediator connecting body weight to chronic illness.

In sum, this research indicates that an effective approach to weight management for older adults may need to be holistic and context-aware. These findings support the call for the development of differentiated health strategies that move beyond a singular focus on BMI to integrate psychological well-being and account for the distinct challenges and resources present in urban and rural settings. Ultimately, these insights provide a robust evidence base to inform more precise and humane health policies aimed at promoting healthy aging within the framework of the “Healthy China 2030” initiative.

## Data Availability

The original contributions presented in the study are included in the article/supplementary material, further inquiries can be directed to the corresponding author.
